# High *OCT4* and Low p16^INK4A^ Expressions Determine *In Vitro* Lifespan of Mesenchymal Stem Cells

**DOI:** 10.1155/2015/369828

**Published:** 2015-05-21

**Authors:** Carla A. Piccinato, Andrea L. Sertie, Natália Torres, Mario Ferretti, Eliane Antonioli

**Affiliations:** ^1^Hospital Israelita Albert Einstein, 627/701, 05652-900 São Paulo, SP, Brazil; ^2^Orthopedic Division, Federal University of Sao Paulo, 783, 04038-031 São Paulo, SP, Brazil

## Abstract

After long-term culture, mesenchymal stem cells alter their biological properties and enter into a state of replicative senescence. Although several classical biomarkers have been used for quantitative assessment of cellular senescence, no hallmark has been proven completely unique to the senescent state in cells. We used bone marrow-derived MSCs (BM-MSCs) from different healthy young donors and an *in vitro* model with well-defined senescence end points to identify a set of robust markers that could potentially predict the expansion capacity of MSCs preparations before reaching senescence. For each early passage BM-MSC sample (5th or 6th passages), the normalized protein expression levels of senescence-associated markers p16^INK4A^, p21^WAF1^, SOD2, and rpS6^S240/244^; the concentration of IL6 and IL8 in cell culture supernatants; and the normalized gene expression levels of pluripotency markers *OCT4*, *NANOG*, and *SOX2* were correlated with final population doubling (PD) number. We revealed that the low expression of p16^INK4A^ protein and a high *OCT4* gene expression, rather than other evaluated markers, might be potential hallmarks and predictors of greater *in vitro* lifespan and growth potential, factors that can impact the successful therapeutic use of MSCs preparations.

## 1. Introduction

Mesenchymal stem cells (MSCs) have the ability to self-renew and differentiate into diverse mesodermal lineages like osteocytes, adipocytes, and chondrocytes. An increasing number of studies have indicated that MSCs from different origins represent a promising source of cells for regenerative medicine. However, due to the low frequency at which MSCs occur in human tissues, extensive* in vitro* expansion is usually required to obtain a sufficient number of cells for clinical applications. It is well known that after long-term culture and sequential passages* in vitro*, MSCs, as most adult cells, alter their biological properties and enter into a state of replicative senescence [1, 2].

Replicative senescence refers to the essentially irreversible growth arrest that occurs when cells lose their ability to replicate after a certain number of population doublings (PDs) in culture [3]. These senescent cells become enlarged, acquire a flattened and irregular morphology, and display a senescence-associated secretory phenotype (SASP), with increased secretion of cytokines, growth factors, and matrix remodeling enzymes. Those are consequences of shortened telomeres, oxidative stress, DNA damage, epigenomic disruption, and other senescence-inducing stimuli [4]. Currently, it is believed that the physiological relevance of replicative senescence lays on the attempt to suppress cancer and facilitate tissue repair (revised by [5, 6]).

Several biomarkers have been used for quantitative assessment of cell senescence in culture and* in vivo*. The senescence-associated *β*-galactosidase (SA-*β* gal) arose as the first more specific marker for the identification of senescent cells and appears to reflect an increased lysosomal mass during replicative aging [7, 8]. The cyclin-dependent kinase inhibitors p16^INK4A^ and p21^WAF1^, important regulators of the cell cycle, have also been widely used as molecular markers of senescence. These proteins are components of two major tumor suppressor pathways, p16^INK4A^/pRb and p53/p21^WAF1^, involved in the control of permanent growth arrest in senescent cells [9, 10]. Studies of aging showed increased expression of p16^INK4A^ with chronological age or PDs of cells in culture. However, p16^INK4A^ is expressed by many, but not all, senescent cells (revised by [11]). Indeed, some senescent states seem to be primarily determined by p16^INK4A^ and others by p53, depending on the need of coupling arrested cell proliferation to other cellular responses, to meet a variety of physiological needs and respond to various forms of stress [5].

Other classical measurable markers that are associated with the senescent state include upregulated secretion of the proinflammatory molecules interleukin-6 and interleukin-8 (IL6 and IL8), key components of the SASP, which actively participate in the senescence process and reinforce the senescent growth arrest (revised by [12]). Additionally, there are many important but less commonly used markers to assess senescence, including proteins involved in oxidative stress responses, such as the antioxidant enzyme SOD2 whose downregulation induces mitochondrial oxidative stress [13]. Also, components of the mTOR signaling pathway, such as the ribosomal protein rpS6^S240/244^ (activated at Ser240/244 sites as part of the signal transduction process), have been recently shown to play essential roles in cell senescence and organismal aging [14–16].

Senescence-associated markers are usually not restricted to senescent passages but are continuously acquired since the beginning of the* in vitro* culture [1], revised by [17]. Moreover, no marker or hallmark has been proven completely unique to the senescent state in cells, and not all senescent cells express all senescence markers. Thus, alternative markers would be useful in the assessment of cellular senescence. Because they exhibit an opposite biological function compared to senescence, downregulated expression of pluripotency-related transcription factors may be pointed as a potential candidate marker of the senescent state of cells, especially for stem cells. Pluripotency genes, such as* OCT4* and* NANOG*, have shown to be expressed in both pluripotent and adult stem cells (such as MSCs) and are downregulated upon long-term* in vitro* expansion and differentiation [18, 19]. It remains to be elucidated, however, whether coexpression of senescence- and pluripotency-related factors affect the lifespan of MSCs and, therefore, their cellular performance and effectiveness in therapy.

It is known that differences in the long-term proliferative capability of MSCs isolated from different donors exist [1, 20] and, importantly, the* in vitro* expansion potential of different MSCs samples may be related to morphological and molecular characteristics expressed in early passages. In order to further explore this hypothesis, the present study aimed to examine how the early expression of senescence markers, p16^INK4A^, p21^WAF-1^, IL6, IL8, SOD2, and rpS6^S240/244^, and pluripotency markers,* OCT4*,* SOX2*, and* NANOG*, would help to predict the ability of MSCs to long-term proliferate in culture before entering a growth arrest typical of senescence. We used bone marrow-derived MSCs (BM-MSCs) from different healthy young donors and an* in vitro* model with well-defined senescence end points to investigate this important issue.

## 2. Materials and Methods

### 2.1. Cell Culture

Primary human MSCs isolated from bone marrow of healthy young adults (3 males and 3 females, aging 21–39 years old) were obtained from patients undergoing anterior cruciate ligament (LCA) reconstruction surgery. The study was carried out in full accordance with ethics guidelines, and samples were collected after written informed consent was obtained.

For MSCs isolation, a small volume of bone marrow was aspirated from distal femur, diluted with equal volume of PBS (phosphate buffered saline), and then overlaid on Ficoll-Paque (density = 1.03 to 1.12 g/mL; GE Healthcare) for density gradient separation by centrifugation at 500 g for 30 minutes. The mononuclear cells were collected and cultured in DMEM-low glucose (Gibco/Life Technology) supplemented with 15% fetal bovine serum (Gibco/Life Technology), 1 mM L-glutamine, and 1% antibiotic-antimycotic solution (Gibco/Life Technology) in T-25 flasks and were incubated at 37°C in a humidified 5% CO_2_. Nonadherent cells were removed after 24 h, and the media were replaced 3 times a week until cells reached 70–80% confluence. The cells were harvested with trypsin/EDTA solution (0.25% trypsin, 4 mM EDTA; Gibco/Life Technology), and cell number and viability were assessed by the Trypan Blue exclusion method. At every passage, cells were seeded at a density of 4000 cells/cm^2^.

The number of PDs was calculated based on the total cell number at each passage using the following equation:

(1) $$\begin{array}{c} \text{cumulative PD}=\frac{\log_{10}\left(NH/NI\right)}{\log_{10}\left(2\right)}, \end{array}$$

where PD is population doublings, NH is cell harvest number, and NI is plating cell number.

### 2.2. Characterization of Bone Marrow Mesenchymal Stem Cells

BM-MSCs were expanded until the fifth passage and analyzed by flow cytometry to determine the expression profile of stem cell markers as defined by the International Society of Cell Therapy. All BM-MSCs samples were found to be positive for CD90, CD73, and CD105 (>95% positive cells) and negative for CD45, CD34, CD14, and HLA-DR. Data were collected using FACSAria (BD Bioscience) and analyzed using FlowJo software (Tree Star, Inc., Ashland, OR). Differentiation of BM-MSCs into adipocytes, osteocytes, and chondrocytes was successfully achieved by using specific media (StemPro Adipogenesis, Chondrogenesis and Osteogenesis Differentiation kit, Gibco/Life Technologies) and confirmed using lineage-specific cell staining: Oil Red for adipocytes, Alcian Blue for chondrocytes, and Alizarin Red S for osteocytes.

### 2.3. Senescence-Associated-*β*-Galactosidase Assay

Cellular senescence was assessed using the *β*-galactosidase staining kit (Cell Signaling) following the manufacturer's instruction. In brief, cells were seeded in 48-well plates and cultivated for 24 hours. After washing with PBS, cells were fixed with 4% formaldehyde in PBS for 15 min and stained for *β*-galactosidase activity using X-gal solution (pH 6.0) at 37°C overnight. Cells were analyzed under a phase-contrast microscope. To discriminate morphology, cells were also stained with hematoxylin.

### 2.4. Immunoblotting

Total protein extracts from cultured cells were obtained using Ripa Buffer (50 mM TrisHCl pH 7.4, 150 mM NaCl, 2 mM EDTA, 1% NP-40, and 0.1% SDS) containing protease and phosphatase inhibitor cocktails (Sigma). Equal amounts of proteins (20 *μ*g) were separated by SDS-PAGE and transferred to nitrocellulose membranes by western blotting. Membranes were then blocked and incubated with the following primary antibodies: anti-P16 (ab108349, Abcam), anti-P21 (ab109199), anti-SOD2 (sc-30080, Santa Cruz Biotechnology), anti-phospho-rpS6^S240/244^ (number 5364, Cell Signaling Technology), and B-actin (A2228, Sigma) for loading control. Detection was performed using horseradish peroxidase-coupled anti-mouse or anti-rabbit secondary antibodies (Cell Signaling), ECL substrate (GE Healthcare), and conventional developing using X-ray films. The intensity of the bands was determined by densitometry using Totalab TL120 software (Nonlinear Dynamics, Newcastle upon Tyne, United Kingdom).

### 2.5. Inflammatory Cytokines Measurement

The levels of inflammatory molecules IL6 and IL8 in culture supernatants were evaluated using the Cytometric Bead Array (CBA) Human Inflammatory Cytokine Kit (BD Biosciences) by flow cytometry following the manufacturer's instructions. Data were analyzed using FCAP Array software (Soft Flow Inc.).

### 2.6. Gene Expression Analyses

Total RNA was extracted from cultured cells using the Illustra RNAspin Mini RNA isolation kit (GE Healthcare), and reverse transcription of 1 *μ*g extracted RNA was performed with QuantiTect Reverse Transcription kit (QIAGEN). qRT-PCR was carried out in an ABI7500 thermocycler (Applied Biosystems, Carlsbad, California) using the Maxima SYBR Green qPCR Master Mix (Thermo Fisher Scientific Inc., Waltham, MA), according to the manufacturer's recommendations. Gene-specific primers were designed using Primer Express software (version 1.5; Applied Biosystems, Foster City, CA) and are listed in Table 1. The genes of interest that were analyzed by qRT-PCR included* OCT4*,* NANOG*, and* SOX2*. The expression level of each target gene was normalized to the* GAPDH* mRNA level, which was measured concurrently. Results are expressed as the mean fold change of the normalized gene expression relative to a calibrator sample (Reference RNA for Real-Time qPCR, number 636690, Clontech, Mountain View, CA, US), using the ^ΔΔ^Ct method [21].

### 2.7. Statistical Analysis

Statistical analysis was performed with SAS software (SAS Institute, 1998). The evaluation of factors potentially correlated with lifespan, here measured as cumulative PD, was performed by the CORR procedure of SAS, using the Spearman correlation method. For each early passage BM-MSC sample (5th or 6th passages), the normalized protein expression levels of p16^INK4A^, p21^WAF1^, SOD2, and rpS6^S240/244^; the concentration of IL6 and IL8 in cell culture supernatants; and the normalized gene expression levels of* OCT4*,* NANOG*, and* SOX2* were plotted against the final PD number (senescent passage). In all analysis, the level of significance was considered *P* < 0.05.

## 3. Results

### 3.1. Intersubject Variability in BM-MSCs Morphology and Long-Term Growth

**Figure 1:**
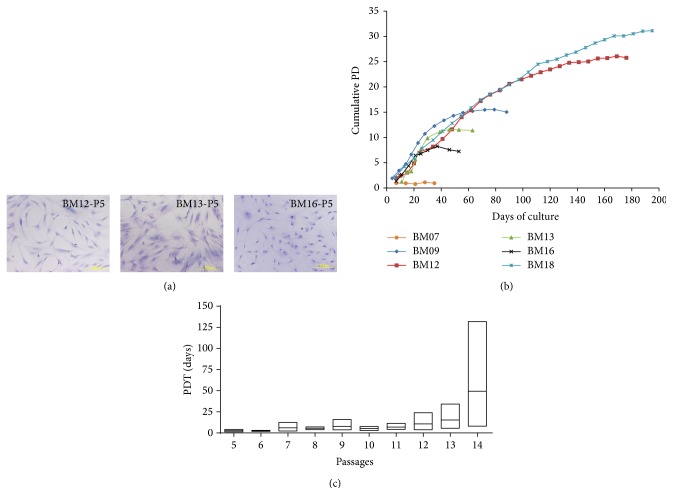
Morphological features and growth kinetics of BM-MSCs. (a) Representative images of BM-MSCs at an early passage (5th passage) showing that different morphology was observed in cells from different donors, ranging from a typical elongated and spindle-like shape (sample BM12) to an enlarged and irregular morphology (samples BM13 and BM16). Cells were stained with hematoxylin. Scale bar: 200 *μ*m. (b) Cumulative population doublings (PD) of BM-MSCs from 6 young healthy donors until replicative senescence (growth arrest). Cell numbers were determined at every passage and cumulative PD was calculated. Each line represents BM-MSCs from one donor and each symbol represents one passage. (c) The mean PD time (PDT) of multiple donors at each passage, showing increased interindividual growth variability and greater PDT over time.

BM-MSCs were obtained from six healthy young donors (referred to as samples BM07, 09, 12, 13, 16, and 18). At passages 5 to 6, an immunophenotypically homogeneous population of BM-MSCs (CD73^+^, CD90^+^, CD105^+^, CD45^−^, CD34^−^, CD14^−^, and HLA-DR^−^) with adipogenic, osteogenic, and chondrogenic differentiation potential was observed for all samples (data not shown). However, at these early passages, we observed that BM-MSCs from different donors presented distinct morphology, ranging from a typical elongated and spindle-like morphology (samples BM09, 12, and 18) to an irregular appearance with a larger shape, compatible with cells entering senescence (samples BM07, BM13, and BM16) (Figure 1(a)).

The proliferation potential of BM-MSCs cultures was measured by cumulative PD level, ranging from 12 to 32 cumulative PDs (Figure 1(b)). Population doubling curves varied considerably among cultures. Similarly, time to reach senescence differed between cultures. Among the cultures evaluated in the present study, BM16 and BM13 stopped to proliferate in early passages with 11.8 and 8.8 PDs, respectively. Intriguingly, BM07 never completed a single PD (0.95), suggesting it was already showing senescence signs even in early passages. In contrast, BM12 and BM18 took longer time to achieve senescence, reaching more than 25 PDs. The time course experiments showed that proliferation declined in time before entering a growth arrest typical of senescence. Although cells were seeded at the same density, there was also a considerable variability in the population doubling time (PDT) among cultures. Overall, PDT variability was lower in early passages as compared to latter passages. Also, PDT increased with the time course, showing a slower growth rate after latter passages (Figure 1(c)).

### 3.2. Culture Expansion of BM-MSCs Causes Variable Changes in the Morphology and Expression Patterns of Classical Senescence-Associated Markers

To characterize phenotypic and molecular changes in BM-MSCs associated with senescence, we analyzed the cellular morphology and the expression patterns of classical senescence-associated markers, *β*-galactosidase, p16^INK4A^, p21^WAF1^, IL6, and IL8, in BM-MSCs at an early passage (P5) and at the senescent (last) passage.

**Figure 2:**
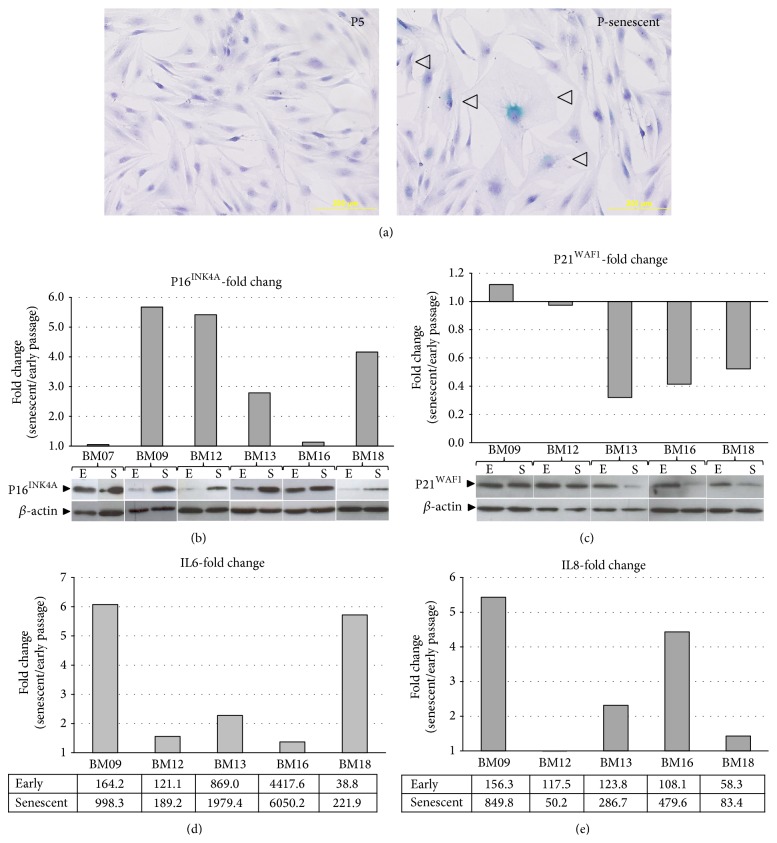
Morphological and molecular changes of senescent BM-MSCs. (a) Representative images of cells at early passage (5th passage) and late/senescent passage. Most early passage cells showed spindle-like morphology and few cells stained positive for the senescence-associated *β*-galactosidase marker. Most senescent passage cells showed enlarged and flattened morphology and an increased number of cells stained for *β*-galactosidase (arrows). (b, c) The protein expression levels of p16^INK4A^ and p21^WAF1^ in early and late passage cells from the same donor were analyzed by western blot. Band intensities were densitometrically evaluated and p16^INK4A^ and p21^WAF1^ protein levels were quantified and normalized to the corresponding *β*-actin levels (loading control). Subsequently, the normalized values obtained were then used to calculate the fold change in expression between late and early passage cells (bar graphs). These results are representative of three independent experiments. Most senescent cells showed increased p16^INK4A^ expression levels. On the contrary, most senescent cells showed decreased p21^WAF1^ expression levels. (d, e) The levels of secreted IL6 and IL8 in early and late passage cells from the same donor were determined using a CBA proinflammatory kit. Values obtained (expressed as pg/mL, bottom) were then used to calculate the fold change in secreted protein levels between late and early passage cells (bar graphs). These results were obtained in triplicate and are representative of two independent experiments. Most senescent BM-MSCs showed enhanced secretion of these proinflammatory cytokines.

We observed that BM-MSCs replicative senescence was accompanied by typical morphological changes and cells became enlarged and flattened with increased cytoplasmic granularity and enhanced *β*-galactosidase activity (Figure 2(a)). However, these senescence-associated alterations were more pronounced in BM-MSCs samples that showed relatively longer* in vitro* lifespan (BM09, BM12, and BM18), suggesting that samples that presented senescence-like morphology at early passages and reduced PD potential might be already senescent.

Most senescent BM-MSCs also showed increased p16^INK4A^ protein expression as compared to early passage BM-MSCs from the same donor (Figure 2(b)). On the other hand, we observed a decrease in p21^WAF1^ protein expression, another CDK inhibitor, in most senescent BM-MSCs compared with early passage cells from the same donor (Figure 2(c)). Curiously, the divergent expression level of these two proteins, p16^INK4A^ and p21^WAF1^, presented a strong and inverse relationship between fold changes (senescent/early passage) (*r* = 0.9, *P* = 0.037), which may suggest that at senescence of BM-MSCs they are expressed in opposite directions. In addition, most senescent BM-MSCs also showed enhanced secretion of the proinflammatory cytokines IL6 and IL8 (Figures 2(d)-2(e)). Finally, it is noteworthy for all proteins measured that there is interindividual variability in the fold change (senescent/young passage) of expression levels.

### 3.3. p16^INK4A^ and OCT4 Are the Most Robust Lifespan Markers in Early Cultured BM-MSCs

In order to identify easily measurable factors that may be predictive of BM-MSCs growth potential, our strategy was to quantify the expression levels of already established markers of senescence and pluripotency at early passages (5th or 6th passages) of each BM-MSC sample and correlate them with the final numbers of PDs.

**Figure 3:**
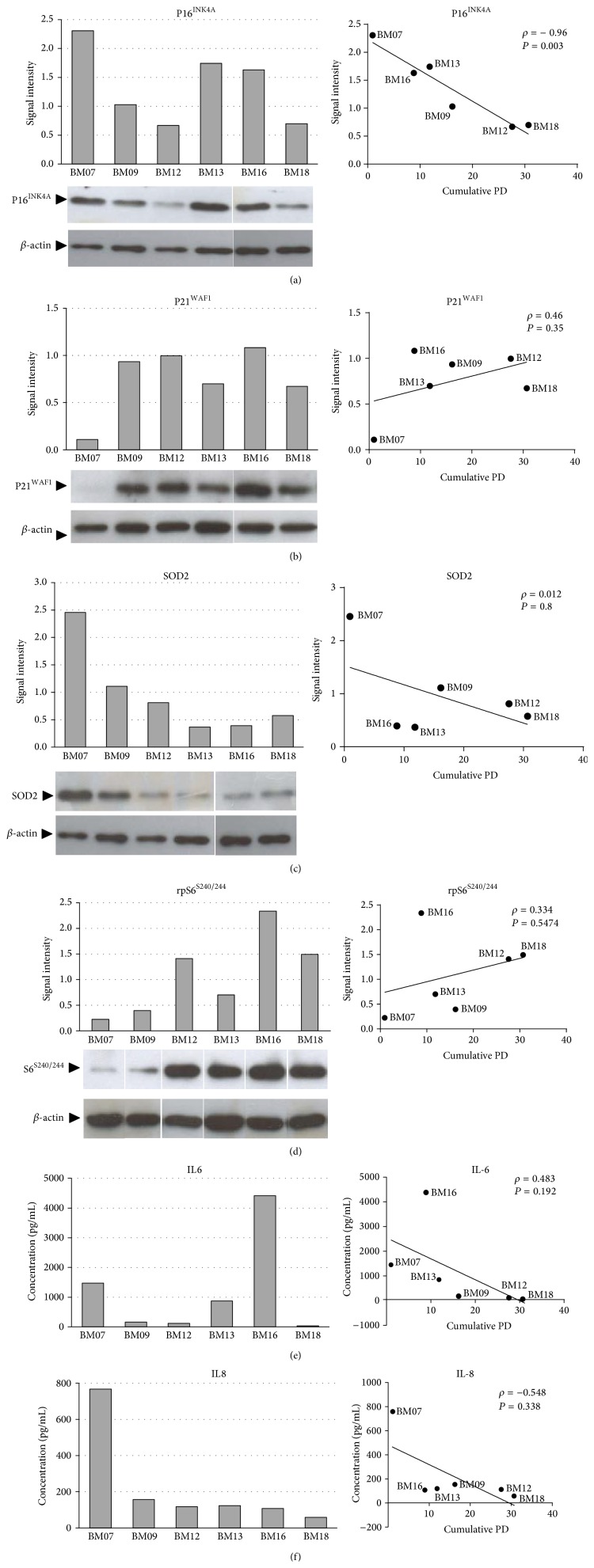
Low p16^INK4A^ protein expression as a robust early marker of BM-MSC lifespan. The protein expression levels of (a) p16^INK4A^, (b) p21^WAF1^, (c) SOD2, and (d) rpS6^S240/244^ in early passage cells from each donor were analyzed by western blot. Band intensities were densitometrically evaluated and bar graphs on the left represent the densitometric values of p16^INK4A^, p21^WAF1^, SOD2, and rpS6^S240/244^ normalized to the loading control (*β*-actin). Normalized expression values were then plotted against the final PD number and statistically analyzed by Spearman correlation, as shown in the graphs on the right. The expression levels of p16^INK4A^ protein in early passage cells were inversely correlated with lifespan. Results are representative of three independent experiments, with similar results.

Among the senescence markers, p16^INK4A^ protein expression was significant and inversely correlated with lifespan (*r* = −0.89, *P* = 0.018) (Figure 3(a)), suggesting that cells with relatively lower expression levels of this protein at early passages present an extended replicative potential. Other senescence-related proteins such as p21^WAF1^, SOD2, and rpS6^S240/244^ were not significantly correlated with lifespan (Figures 3(b)–3(d)). Similarly, the amount of IL6 and IL8 secreted at early passages could not correlate with the growth capacity of BM-MSCs (Figures 3(e)-3(f)).

**Figure 4:**
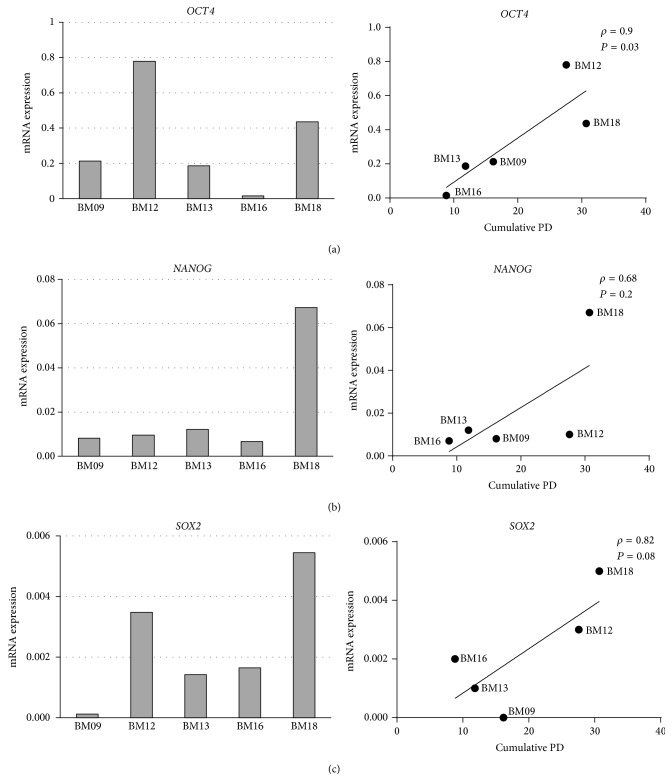
High* OCT4* gene expression as a robust early marker of BM-MSC lifespan. The gene expression levels of (a)* OCT4*, (b)* NANOG*, and (c)* SOX2* in early passage cells from each donor were analyzed by real time PCR. Relative gene expression levels were quantified using the ΔΔCt method and the results were normalized to the expression of GAPDH. Bar graphs on the left represent the mean fold change of the normalized gene expression relative to a calibrator sample (reference RNA). These values were then plotted against the final PD number and statistically analyzed by Spearman correlation, as shown in the graphs on the right. The expression levels of* OCT4* in early passage cells were positively correlated with lifespan. Results are representative of two independent experiments, each performed in duplicate, with similar results.

Due to low protein expression of pluripotency markers (*NANOG*,* OCT4*, and also* SOX2*) in BM-MSCs, we could not evaluate the ability of such proteins in estimating lifespan. Since gene expression techniques might be more sensitive, we proposed to measure mRNA abundance of pluripotency-related genes by real time PCR. We observed that the expression of* OCT4* showed strong and positive correlation with lifespan (*r* = 0.9, *P* = 0.037) (Figure 4(a)), indicating that cells with more abundant expression of such pluripotency gene at early passages could potentially present an extended lifespan. The expression of other evaluated pluripotency-related genes,* NANOG* and* SOX2*, did not present a significant correlation with cellular longevity (Figures 4(b)-4(c)).

Because the* OCT4*/p16^INK4A^ ratio was directly correlated with lifespan (*r* = 0.9, *P* = 0.037), our results suggest that a lower p16^INK4A^ and a higher* OCT4* level of expression work as a great hallmark to estimate the senescent state of MSCs preparations and therefore may be good predictors of their* in vitro* expandability.

## 4. Discussion

Culture-expanded human MSCs are increasingly used for both research and clinical purposes. Morphological analysis, evaluation of specific cell surface markers, and differentiation into mesenchymal lineages are primarily useful to characterize MSCs expanded* in vitro*, according to the minimum criteria established by the International Society for Cell Therapy [22]. There is no guarantee, however, that the characterized MSCs samples would perform well during* in vitro* expansion and* in vivo* cell transplantation. Therefore, it would be of utmost interest to identify a set of robust markers that predict the expansion capacity of MSCs preparations before reaching senescence, which can be valuable information when large numbers of MSCs are required. The present study intended to improve knowledge in this area.

Herein, we used a robust model of replicative senescence with well-defined endpoints. Yet the used BM-MSCs samples showed high interindividual variability in morphology, long-term growth profile, and time to reach senescence, which was similar to what was described in previous studies [23–26]. Notably, no clinically relevant differences were observed among donors and BM-MSCs were isolated, cultivated, and expanded under the same standardized controlled conditions. Nevertheless, inconsistencies during such procedures could play a role in the described variability and thus cannot be ruled out. Alternatively, reprogramming upon removal from the* in vivo* marrow niche [27], different clonal populations [28], and alterations due to high oxygen species accumulation and methylation [29] might also explain the observed morphological and functional heterogeneity.

Taking advantage of such interindividual variability in the replicative lifespan of our BM-MSCs samples, this study focused on understanding key factors that could predict* in vitro* long-term growth potential of MSCs preparations. Our strategy was to find markers whose expression levels at early passages were correlated with lifespan and with the senescent status of the cells. Initially, by examining established markers of senescence, we observed, in agreement with previous studies [30, 31], that the expression levels of p16^INK4A^ protein increase in senescent BM-MSCs and, more importantly, that its expression in early passage cells shows a strong and negative correlation with the final number of PDs. The association of p16^INK4A^ and cellular lifespan seems to be due to the direct role of the pRB pathway in establishing and/or maintaining growth arrest and, consequently, anchoring the cells in a senescent state [32]. In fact, knockdown of p16^INK4A^ *in vitro* has proven to reverse senescent behavior of MSCs in different models [19, 30, 31]. These results suggest that although senescence is a dynamic process, cells with greater p16^INK4A^ expression might be closer to the replicative senescent phase.

On the other hand, we observed that the expression levels of p21^WAF1^ protein, downstream effector of the p53 growth arrest pathway, reduce in senescent BM-MSCs and that its expression in early passage cells is not correlated with the growth potential of these cells. Interestingly, we observed a tight and inverse relationship between fold changes (senescent/early passage) of p16^INK4A^ and p21^WAF1^. The rationale for this result is the observation that the activation of p21^WAF1^ during the senescence process in some cells is only transient with levels declining after growth arrest is established, whereas p16^INK4A^ is responsible for the maintenance of growth arrest in the senescent cells [32]. Although so far there are no senescence markers that are capable of unequivocally identifying senescent cells either* in vitro* or* in vivo*, our results and those of previous studies indicate that p16^INK4A^, but not p21^WAF1^, might be a better marker of the senescent state of MSCs [5, 30].

Although we observed enhanced release of the proinflammatory cytokines IL6 and IL8 in most senescent cells as compared to early passage cells from the same donor, we did not detect any significant correlation between IL6 or IL8 levels in supernatants from early passage cells and lifespan. These results suggest, in agreement with previous studies, that acquisition of a SASP is a later event in the senescence process that acts to reinforce the senescence growth arrest in an autocrine manner [33] revised by [12, 34].

A similar lack of correlation was observed between BM-MSCs lifespan and the protein expression levels of the antioxidant enzyme SOD2 or the ribosomal protein rpS6^S240/244^ in cells at early passage numbers. Recent studies have suggested that increased mitochondrial oxidative stress and mTOR signaling pathway are involved in cellular senescence, including senescence of MSCs [16, 35]. Although it was outside the scope of the current study to further explore these biological processes, our results suggest that, as observed for IL6 and IL8, the expression levels of SOD2 and rpS6^S240/244^ in MSCs at early passages are not predictive of their expandability potential.

Besides their role in maintaining the undifferentiated status of pluripotent stem cells, pluripotency-associated genes have been also related to the regulation of stem cells properties of MSCs [19, 36]. Our results showed that MSCs with relatively high* OCT4* gene expression presented an increased lifespan. In addition, a high* OCT4*/p16^INK4A^ ratio was correlated with increased lifespan and, as far as we know, this is the first study that demonstrates a direct association of a pluripotency gene and its combination with p16^INK4A^, with cellular longevity and senescence. Moreover, we also identified a strong inverse correlation between early expression of* OCT4* and p16^INK4A^ in our samples. This is not unexpected given that an increased expression of key pluripotency genes downregulates cell cycle regulators such as p16^INK4A^ and p21^WAF1^ [19]. The mechanisms that underlie this observation involve direct binding of OCT4 and NANOG to the promoter of DNA methyltransferase 1, which enhances its expression and, in turn, maintains DNA methylation and downregulates p16^INK4A^ and p21^WAF1^ expression, thus regulating the proliferative and undifferentiated status of MSCs [19].

Our data suggest that low levels of p16^INK4A^ and high levels of* OCT4* may predict the senescent state and the proliferative capacity of early passage MSCs. Because replicative senescence also reduces the differentiation capacity of MSCs [37], in future studies, it will be interesting to address whether the combined expression of p16^INK4A^ and* OCT4* in opposite directions would also be the best predictor of the multipotency of individual MSC preparations.

## 5. Conclusion

Taken together, our results further corroborate and extend previous findings suggesting that (1) there is high variability in early morphology and long-term proliferative capability of MSCs isolated from different donors; (2) lower expression of p16^INK4A^, but not p21^WAF1^, might be a better marker of the senescent state of MSCs; (3) expression levels of IL-6, IL-8, SOD2, rpS6^S240/244^,* SOX2*, and* NANOG* at early cell passages do not correlate with MSCs lifespan; (4) MSCs express the pluripotency-associated gene* OCT4* and its expression is related to the proliferation capacity of these cells. In conclusion, an early lower p16^INK4A^ and a higher* OCT4* level of expression work as a great hallmark to estimate the senescent state of MSCs and to predict their* in vitro* expandability.

## Figures and Tables

**Table 1:** Sequence of primers and probes.

Gene name	Sequence
GAPDH	5′-ATTGCCCTCAACGACCACTT-3′
	5′-TGCTGTAGCCAAATTCGTTGTC-3′
OCT4	5′-CCTCACTTCACTGCACTGTA-3′
	5′-CAGGTTTTCTTTCCCTAGCT-3′
SOX2	5′-CCATCCACACTCACGCAAAA-3′
	5′-TATACAAGGTCCATTCCCCCG-3′
NANOG	5′-TGGACACTGGCTGAATCCTTC-3′
	5′-CGTTGATTAGGCTCCAACCAT-3′
